# Using Organic Contaminants to Constrain the Terrestrial Journey of the Martian Meteorite Lafayette

**DOI:** 10.1089/ast.2021.0180

**Published:** 2022-10-31

**Authors:** Áine Clare O'Brien, Lydia Jane Hallis, Clement Regnault, Douglas Morrison, Gavin Blackburn, Andrew Steele, Luke Daly, Alastair Tait, Marissa Marie Tremblay, Darcy E.P. Telenko, Jacqueline Gunn, Eleanor McKay, Nicola Mari, Mohammad Ali Salik, Philippa Ascough, Jaime Toney, Sammy Griffin, Phil Whitfield, Martin Lee

**Affiliations:** ^1^ School of Geographical and Earth Sciences, University of Glasgow, Lilybank Gardens, Glasgow, UK.; ^2^ SUERC, University of Glasgow, East Kilbride, UK.; ^3^ Polyomics, University of Glasgow, Wolfson Wohl Cancer Research Centre, Switchback Rd, Bearsden, Glasgow, UK.; ^4^ Carnegie Planets, Carnegie Science, Washington DC, USA.; ^5^ Australian Centre for Microscopy and Microanalysis, The University of Sydney, Sydney, Australia.; ^6^ Department of Materials, University of Oxford, Oxford, UK.; ^7^ School of Earth, Atmosphere & Environment Monash University, Rainforest Walk Clayton, Victoria, Australia.; ^8^ Department of Earth, Atmospheric, and Planetary Sciences, Purdue University, West Lafayette, Indiana, USA.; ^9^ Department of Botany and Plant Pathology, Purdue University, West Lafayette, Indiana, USA.; ^10^ School of Professional Services, Glasgow Caledonian University, Cowcaddens Road, Glasgow, UK.; ^11^ Dipartimento di Scienze della Terra e dell'Ambiente, University of Pavia, Pavia, Italy.

**Keywords:** Lafayette, Mars, Meteorites, Organics, Metabolomics, Fireball

## Abstract

A key part of the search for extraterrestrial life is the detection of organic molecules since these molecules form the basis of all living things on Earth. Instrument suites such as SHERLOC (Scanning Habitable Environments with Raman and Luminescence for Organics and Chemicals) onboard the NASA Perseverance rover and the Mars Organic Molecule Analyzer onboard the future ExoMars Rosalind Franklin rover are designed to detect organic molecules at the martian surface. However, size, mass, and power limitations mean that these instrument suites cannot yet match the instrumental capabilities available in Earth-based laboratories. Until Mars Sample Return, the only martian samples available for study on Earth are martian meteorites. This is a collection of largely basaltic igneous rocks that have been exposed to varying degrees of terrestrial contamination. The low organic molecule abundance within igneous rocks and the expectation of terrestrial contamination make the identification of martian organics within these meteorites highly challenging. The Lafayette martian meteorite exhibits little evidence of terrestrial weathering, potentially making it a good candidate for the detection of martian organics despite uncertainties surrounding its fall history. In this study, we used ultrapure solvents to extract organic matter from triplicate samples of Lafayette and analyzed these extracts via hydrophilic interaction liquid chromatography–mass spectrometry (HILIC-MS). Two hundred twenty-four metabolites (organic molecules) were detected in Lafayette at concentrations more than twice those present in the procedural blanks. In addition, a large number of plant-derived metabolites were putatively identified, the presence of which supports the unconfirmed report that Lafayette fell in a semirural location in Indiana. Remarkably, the putative identification of the mycotoxin deoxynivalenol (or vomitoxin), alongside the report that the collector was possibly a student at Purdue University, can be used to identify the most likely fall year as 1919.

## 1. Introduction

### 1.1. Organic matter in martian samples

Since the Viking missions, the detection of carbon-based molecules has been a critical aim for martian exploration, as the availability of organic matter (OM) is central to the assessment of martian habitability and the possibility of extinct or extant life on Mars (Biemann *et al.,* 1977; Sagan and Lederberg, 1976). In recent years, OM has been successfully detected on Mars (Eigenbrode *et al.,* 2018; Webster *et al.,* 2015) and within martian meteorites (Sephton *et al.,* 2002; Steele *et al.,* 2012). Furthermore, sulfonated compounds known as thiophenes have been found on Mars that correlate with those found in martian meteorites (Eigenbrode *et al.,* 2018; Steele *et al.,* 2018).

Two of the newest generation of Mars rovers (*i.e.*, the NASA Perseverance and European Space Agency [ESA] Rosalind Franklin rovers) are primed for OM detection. The primary analytical techniques leading this exploration are Perseverance's SHERLOC (Scanning Habitable Environments with Raman and Luminescence for Organics and Chemicals) and Rosalind Franklin's Raman laser spectrometer instruments, and mass spectrometry using the Mars Organic Molecule Analyzer instrument suite onboard the Rosalind Franklin rover (Vago *et al.,* 2017). Perseverance will also be caching samples of astrobiological and geological interest for return to Earth in future missions as part of the Mars Sample Return (MSR) campaign. Since the OM present in martian meteorites has been shown to correlate with that found by rovers on Mars (Steele *et al.,* 2018), these meteorites can be used to further characterize martian OM and prepare for MSR by evaluating OM detection techniques.

Martian meteorites are mostly igneous rocks and are a mixture of falls and finds, with varying levels of terrestrial contamination (Udry *et al.,* 2020). Before MSR, martian meteorite data can complement martian surface OM data by allowing for *in situ* analysis at smaller (nano) scales and bulk rock analyses with lower detection limits than those currently achievable via rover instrumentation. Indigenous OM detected in martian meteorites is primarily macromolecular carbon (MMC), consisting largely of polycyclic aromatic hydrocarbons (Steele *et al.,* 2012). Raman spectroscopy shows that this kerogen-like material is present as thin envelopes that surround silicate-hosted oxide mineral inclusions in a range of meteorite samples (Steele *et al.,* 2016). The mineralogical associations, chemical composition, and carbon isotope ratios of martian meteorite MMC suggest an abiogenic origin from a reduced martian mantle (Becker *et al.,* 1999; Steele *et al.*, 2012). Hydrogen isotope ratios (D/H) from MMC in the Tissint martian meteorite also support a martian origin, with δ*D* values up to +1183‰ (Lin *et al.,* 2014).

In addition to *in situ* techniques such as Raman spectroscopy, OM in martian meteorites has previously been studied by using bulk chromatography techniques, including gas chromatography–mass spectrometry (GC-MS) (Sephton *et al.,* 2002), liquid chromatography–mass spectrometry (LC-MS) (Callahan *et al.,* 2013), and high-performance liquid chromatography (HPLC) (Glavin *et al.,* 1999). LC-MS has also been applied to study OM in carbonaceous chondrites and ureilites (Glavin *et al.,* 2006; Glavin *et al.,* 2010). Meteoritic samples are crushed, dissolved, and in some cases derivatized according to any classes/functional groups targeted, depending on the technique used (Simkus *et al.,* 2019). These techniques can identify individual organic molecules.

However, the challenge lies in separating molecules of martian origin from terrestrial contamination even within meteorites that were collected soon after (days-weeks) they were observed to fall to Earth. For example, a 4-year study of the microbial interactions within the Nakhla martian meteorite fall showed the continual growth and decay of fungal contaminants (Toporski and Steele, 2007). In addition, studies show a progressive change in the D/L ratio of enantiomers in amino acids within the Murchison carbonaceous chondrite since its fall and recovery in 1969 (Glavin *et al.,* 1999; Kvenvolden *et al.,* 1971). These microbial and molecular changes suggest that these meteorites have had significant biogenic contamination in the decades since they fell to Earth, even within a curatorial environment.

Therefore, studying the persistent and dynamic presence of contaminants in martian samples and being able to separate these contaminants from organics of martian origin are crucial in preparation for MSR, to ensure curation and analyses are not compromised (Toporski and Steele, 2007). The aim of this study was to address these knowledge gaps and to evaluate LC-MS as a technique for identifying contaminants through the analysis of an important martian sample obtained from a terrestrial fall.

### 1.2. Constraining the fall history of Lafayette via terrestrial OM contaminants

This study aims to (1) determine the OM content of the martian meteorite Lafayette, (2) distinguish and separate the OM of martian origin from terrestrial contaminants where possible, and (3) evaluate a nontargeted approach to LC-MS as a possible analytical technique of martian samples upon MSR.

Identifying terrestrial OM contaminants within Lafayette will help constrain the meteorite's currently unconfirmed fall history. Nininger (1935) reported that an unidentified Purdue University student was fishing in a pond in Tippecanoe County at an unknown date when the “stone which resembled a corn pone” fell into the mud nearby. The student retrieved the stone and kept it at his home before donating it to Purdue University. Initially, the meteorite was curated within Purdue's geological collection, as the fusion crust re-entry flow markings were mistaken for terrestrial quaternary glacial striations. It was not until 1931 that O.C. Farrington identified Lafayette as a meteorite (Nininger, 1935). Nininger (1935) suggested that the meteorite had not been resting for long on the terrestrial surface before it was picked up since its fusion crust remained largely intact, with minimal weathering features. Regrettably, Farrington passed away before publishing his article on the meteorite, and his notes were never found, meaning that the fall scenario remains unconfirmed.

Lafayette is now classified as a member of the martian nakhlite meteorite group. The nakhlites, which are hypothesized to have originated from a single ejecta crater on Mars due to their ejection ages of 11 Ma (Treiman, 2005), represent a series of basaltic igneous rocks that encompass at least four different magmatic events from a related parental melt that span at least ∼1.4 to 1.3 Ma ago (Cohen *et al.,* 2017). Aqueous alteration from martian fluids, a notable feature across the group, was dated at ∼633 ± 23 Ma within Lafayette (Borg and Drake, 2005). Of the nakhlites, Lafayette contains the most evidence of martian aqueous alteration, identified as veins of “iddingsite” within olivine phenocrysts and matrix regions (Bunch and Reid, 1975). The relationship between shock deformation features and fluid alteration veins within several nakhlite samples, including Lafayette, has led to the current hypothesis of impact-induced hydrothermal activity as the driving mechanism behind alteration (Daly *et al.,* 2019).

## 2. Materials and Methods

### 2.1. A nontargeted LC-MS approach

Metabolomics is the name given to analytical techniques used to detect the metabolic compliment of a system (such as lipids, amino acids, and proteins) in biological samples to identify the presence of certain metabolic pathways (Seyler *et al.,* 2020). These techniques can also be applied to environmental samples to identify endogenous organic compounds and likely contamination compounds (Callahan *et al.,* 2013). There are two main approaches to a metabolomics study, nontargeted and targeted. Targeted analyses involve analyzing specific molecules, or panels of molecules, that are expected to be present within a sample using a specific assay designed to maximize their detection.

Such approaches allow for the accurate identification of compounds should multiple exact mass isomers of the same compound exist. However, this targeted approach requires some knowledge of the type of molecules within the sample before analysis and is limited to just those compounds that can be accurately identified using the chosen assay (Forcisi *et al.,* 2013). In a nontargeted approach, a wide range of metabolite classes are studied, and extraction is designed to sample as many compounds of interest that can be detected by the instrument as possible (Forcisi *et al.,* 2013). With extraterrestrial samples, the exact mass and structure of OM within the sample are unknown, so a nontargeted approach to meteoritic OM detection is more suitable (Seyler *et al.,* 2020).

In the present study, nontargeted LC-MS was used to produce a broad detection of all solvent-soluble molecules polar enough to be detected by the instrument without aiming for a particular classification or specific compound. LC-MS allows for the detection of hundreds of organic molecules with a wide range of masses with high sensitivity (∼70–1050 AMU (Atomic mass units) for the protocol outlined below). As discussed above, LC-MS has previously been used to identify OM in martian meteorites and other extraterrestrial samples. For example, amino acids were identified in the martian meteorite Roberts Massif 04262 via LC-MS, following acid- and water-soluble extractions (Callahan *et al.,* 2013).

### 2.2. Samples

The Lafayette sample used for this study was sourced from a 0.7 g interior chip (meteorite sample with no fusion crust present) of this meteorite allocated to L. Hallis by the London Natural History Museum. To aid the separation of martian OM from potential terrestrial contaminants in Lafayette and to evaluate nontargeted LC-MS as a detection technique, we also studied the OM content of three martian analog samples. The first is a martian simulant developed by NASA: JSC Mars-1 (donated by Dr. A. Steele). This material originates from Pu'u nene, Hawai'i, a hillside region between the Mauna Kea and Mauna Loa volcanoes. JSC Mars-1 is a palognitic tephra (a weathering product with a basaltic composition that forms from the interaction of volcanic glass or hot lava with water) (Morris *et al.,* 1993). Spectroscopic studies have determined that JSC Mars-1 is a close analog to martian dust (Allen *et al.,* 1998).

Two additional analog samples were studied, originating from the Sverrefjellet and Sigurdfjellet regions of the Bockfjorden volcanic complex (BVC) in Svalbard (Steele *et al.,* 2007). The BVC area served as a Mars rover analog mission site for many years, as part of NASA and ESAs AMASE missions. These two BVC samples contain MMC rimmed carbonate globules hosted in igneous minerals, resembling those in the Alan Hills 84001 meteorite (Amundsen *et al.,* 2011; Guzman *et al.,* 2020; Rull *et al.,* 2017; Siljeström *et al.,* 2014; Steele *et al.,* 2007). Analysis of these analog samples also provides the opportunity to catalog their organic content for future reference and allows for increased understanding of the detection limits of the analytical protocol.

### 2.3. Solvent extractions

The Lafayette sample was crushed at Durham University with a brand new aluminum mortar and pestle that was cleaned with Milli-Q H_2_O and quartz. The quartz powder was created by hand in a virgin alumina mortar and pestle. This fine quartz powder was only deemed appropriate for use after prior blank checks. No difference was discerned for total high siderophile elements between empty and precleaned high pressure asher vessels (*i.e.*, reagent+clean digestion vessel blank) and precleaned high pressure asher vessels containing 0.5 g aliquots of quartz powder. These vessels were dedicated for low-/basalt-level samples and were twice cleaned before use for Lafayette, as discussed in the work of Mari *et al.* (2019).

The BVC sample chips were crushed at Scottish Universities Environmental Research Centre (SUERC) with a new agate mortar and pestle that was cleaned with 2% DECON solution, ultrapure water, and then acetone in between uses. The NASA JSC Mars-1 regolith simulant was provided as fine-grained material. Blanks (fresh empty vials opened at the stage introduced) were introduced at each step of analysis to pick up any contaminants present, as outlined in Table 1. The samples were always handled using nitrile gloves.

**Table 1.** An Overview of the Samples Studied, with Their Abbreviations Used for Analysis

Sample ID	Description	Solvent extractions carried out
La 01-03	Lafayette (martian meteorite)	Hexane, dichloromethane, methanol
Sv 01-03	Sverrefjellet (martian analog from Svalbard)	Hexane, dichloromethane, methanol
Si 01-03	Sigurdfjellet (martian analog from Svalbard)	Hexane, dichloromethane, methanol
Js 01-03	JSC Mars-1 (NASA Mars simulant from Hawai'i)	Hexane, dichloromethane, methanol
FB D1 01-03	Procedural blank, introduced as empty vial during crushing/weighing of samples	Hexane, dichloromethane, methanol
FB D2 01-03	Procedural blank, introduced at hexane step	Hexane, dichloromethane, methanol
FB D3 01-03	Procedural blank, introduced at DCM step	Dichloromethane and methanol
FB D3 2 04-06	Procedural blank, introduced at methanol step	Methanol
FB D4 01-03	Procedural blank made up of 1:1:1 hexane, methanol, and DCM introduced at pooling and filtering step	Hexane, dichloromethane, methanol

DCM, dichloromethane.

Solvent extractions were all performed in a positive pressure fume hood at SUERC. Glassware and metal tools were wrapped in foil and placed in a furnace overnight at 450°C. All glassware, ceramics, and tools were then washed in 2% DECON clean solution, rinsed with ultrapure water, and then rinsed with acetone in between uses for different samples. After crushing, 3 × 30 mg replicates from each chip were weighed out and placed in polytetrafluoroethylene (PTFE) screw-top vials using tweezers and foil.

Solvent extracts were performed by adding 1 mL of each solvent to each powdered sample with a Gilson pipette (with a new sterile tip each time) and mixing with a vortex for 10 min at room temperature. Hexane was the first solvent added and mixed, then dichloromethane, and finally methanol. These three solvents were used to target a broad range of apolar to moderately polar molecules, as the extracts were analyzed using both LC-MS and GC-MS (GC-MS analysis was unsuccessful). Each solvent was added, mixed, removed, and then placed in a new vial, using fresh pipette tips before the next solvent was added. A total of 300 μL of each solvent extract was then pooled and mixed in a fresh vial, then removed using a hypodermic needle and syringe, and finally filtered using a 0.45 μm filter and placed in a fresh vial.

**FIG. 1.**
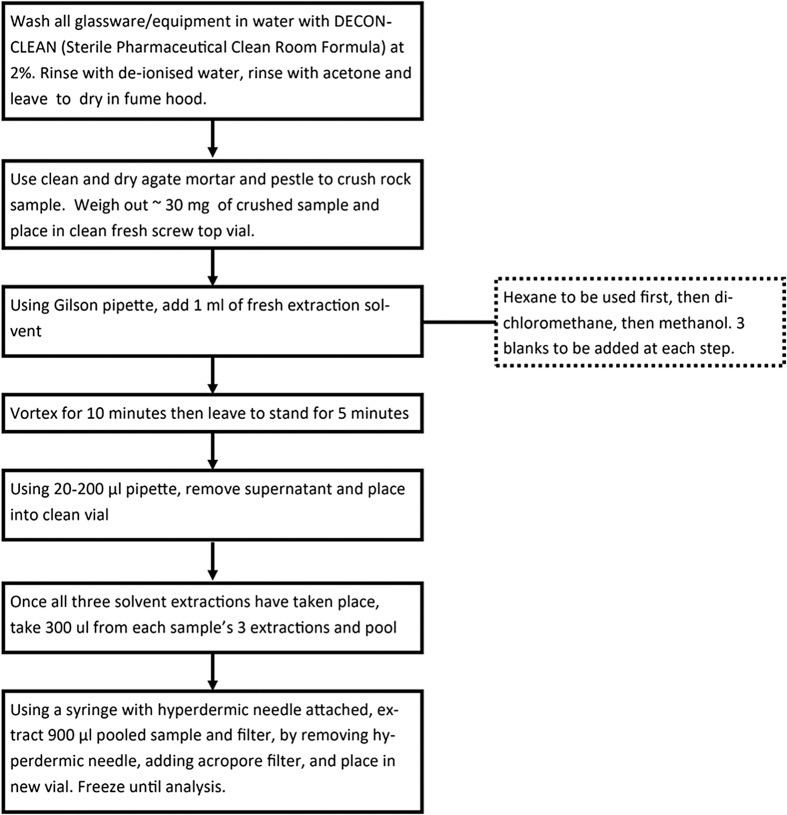
Solvent extraction protocol carried out to capture a broad range of solvent-soluble metabolites in meteorite samples. All steps were carried out at room temperature (∼20°C).

These pooled and filtered extracts were frozen in PTFE screw-top vials at approximately −10°C until LC-MS analysis. Three procedural blanks (fresh vials with solvent added only from that step onward) were added at each new extraction step to identify the introduction of any laboratory contaminants. An overview of this method is given in Fig. 1, and a list of samples studied is given in Table 1.

### 2.4. Liquid chromatography–mass spectrometry

Hydrophilic interaction liquid chromatography (HILIC) was carried out on a Dionex UltiMate 3000 RSLC System (Thermo Fisher Scientific, Hemel Hempstead, United Kingdom) using a ZIC-pHILIC column (150 × 4.6 mm, 5 μm column; Merck Sequant). The column was maintained at 25°C, and samples were eluted with a linear gradient (20 mM ammonium carbonate in water and acetonitrile) over 26 min at a flow rate of 0.3 mL/min as follows:

**Table:** 

Time/min	%A	%B
0	20	80
15	80	20
15	95	5
17	95	5
17	20	80
26	20	80

The injection volume was 10 μL, and samples were maintained at 5°C before injection. For the MS analysis, a Thermo Orbitrap Q Exactive (Thermo Fisher Scientific) was operated in polarity switching mode, and the MS settings were as follows:

- Resolution 70,000
- AGC 1e6
- Mass-to-charge ratio (*m/z*) range 70–1050
- Sheath gas 40
- Auxiliary gas 5
- Sweep gas 1
- Probe temperature 150°C
- Capillary temperature 320°C
- For positive mode ionization: source voltage +3.8 kV, S-lens RF level 30.00, S-lens voltage 25.00 (V), skimmer voltage 15.00 (V), inject flatopole offset 8.00 (V), bent flatapole DC 6.00 (V). For negative mode ionization: source voltage −3.8 kV.

The calibration mass range was extended to cover small metabolites by inclusion of low-mass calibrants with the standard Thermo calmix masses (below *m/z* 138), butylamine (C4H11N1) for positive ion electrospray ionization mode (*m/z* 74.096426), and COF3 for negative ion electrospray ionization mode (*m/z* 84.9906726). To enhance calibration stability, lock-mass correction was also applied to each analytical run shown below:

- Positive Mode Lock masses: Number of Lock Masses: 1 Lock Mass #1 (*m/z*): 144.9822
- Negative Mode Lock masses: Number of Lock Masses: 1 Lock Mass #1 (*m/z*): 100.9856

Instrument .raw files were converted to positive and negative ionization mode mzXML files. These files were processed with IDEOM (Creek *et al.,* 2012; Jankevics *et al.,* 2012), which uses the XCMS (Smith *et al.,* 2006) and mzMatch (Scheltema *et al.,* 2011) software in the R environment. Briefly, this involves using the CentWave algorithm within XCMS to pick out signals based on their retention time and *m/z* ratio. These signals are then grouped based on sample replicates and filtered using relative standard deviation, minimum intensity, and a noise filter to produce a set of signals that are likely to be due to real metabolites.

Finally, a gap-filling step is employed to ensure that signals that may have been missed or lost from a particular group/groups while retained for another group are re-instated, to avoid erroneous identification of signals unique to a particular sample.

Fragmentation data were analyzed in PiMP (Gloaguen *et al.,* 2017) with the FrAnK in-house fragmentation data analysis software. Comparisons between the overall metabolite distributions of the samples were made with MetaboAnalyst (Xia *et al.,* 2009). The list of detected features was further processed using IDEOM (Creek *et al.,* 2012). For metabolite annotation, detected *m/z* ratios were matched against *m/z* in the IDEOM database using a 3 ppm mass error range. A retention time prediction algorithm was then applied to generate a preferred annotation when multiple isomers were present in the database (Creek *et al.,* 2012).

Thus, a drawback of this nontargeted LC-MS approach is that specific molecular identification cannot be verified if multiple isomers of the same exact mass exist—this would require further targeted analysis using standards. The list of putative annotations for all detected LC-MS features can be found in the IDEOM file (Supplementary Materials SM2).

## 3. Results

### 3.1. Comparing Lafayette OM with that of terrestrial analogs

The solvent extraction and LC-MS protocol utilized during this study successfully detected solvent-soluble OM in the Lafayette and terrestrial martian analog samples. Two hundred twenty-four peaks, which could be annotated as likely metabolites (organic molecules detected by the Orbitrap mass spectrometer in the experiments), were detected in Lafayette at intensities more than twice the procedural blanks. Many of the peaks detected in Lafayette were below detection levels in all other samples and blanks, indicating its distinct OM signature.

**FIG. 2.**
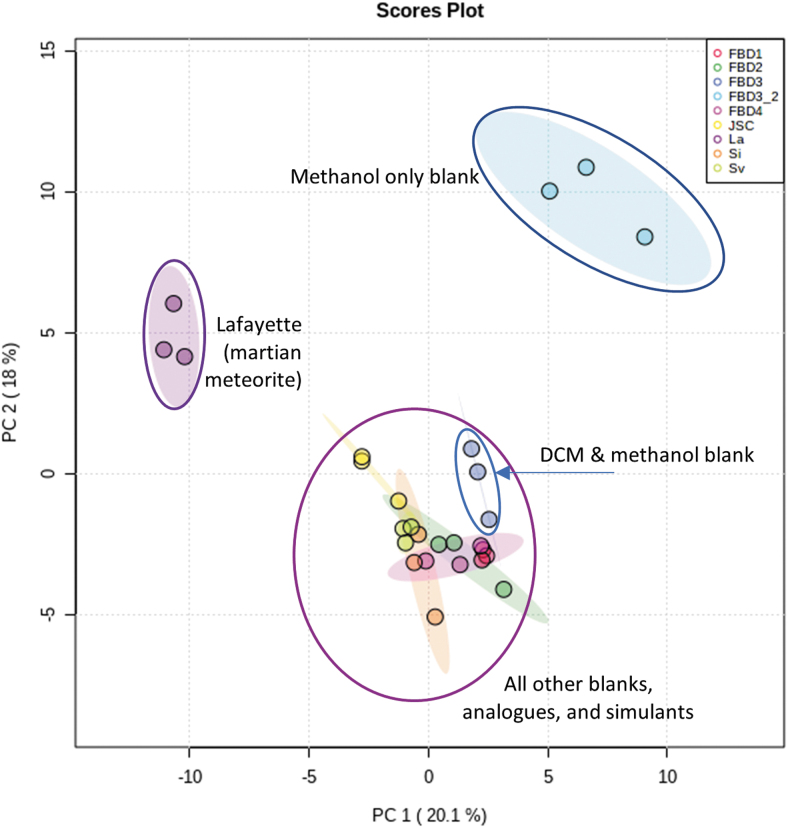
Two-dimensional PCA of LC-MS data from samples of Lafayette, the two BVC analogs, JSC Mars-1, and experimental blanks. PCA shows that the metabolites detected in Lafayette are distinct from those detected in the other samples. The procedural blank made with only methanol is also distinct. PCA was carried out using MetaboAnalyst. BVC, Bockfjorden volcanic complex; LC-MS, liquid chromatography–mass spectrometry; PCA, principal component analysis.

Two-dimensional principal component analysis (PCA) was carried out with MetaboAnalyst (Fig. 2), an open-source metabolomics data analysis tool (Xia *et al.,* 2009). The three Lafayette replicates cluster together, separate from all other samples. Most procedural blanks cluster together, alongside the analog samples. The clearest exceptions to this analog and blanks cluster are the procedural blanks made up of methanol only. The metabolite makeup of the methanol-only blanks is distinct due to high concentrations of molecules with a similar polarity to methanol and low concentrations of less polar molecules that dissolve in hexane, for example. A similar, but less distinct, separation is observable for the blanks without hexane, which cluster slightly apart from the analogs and three-solvent blanks (Fig. 2). This finding illustrates why a three-solvent extraction protocol is required to detect a wide spectrum of solvent-soluble OM within geological samples.

**FIG. 3.**
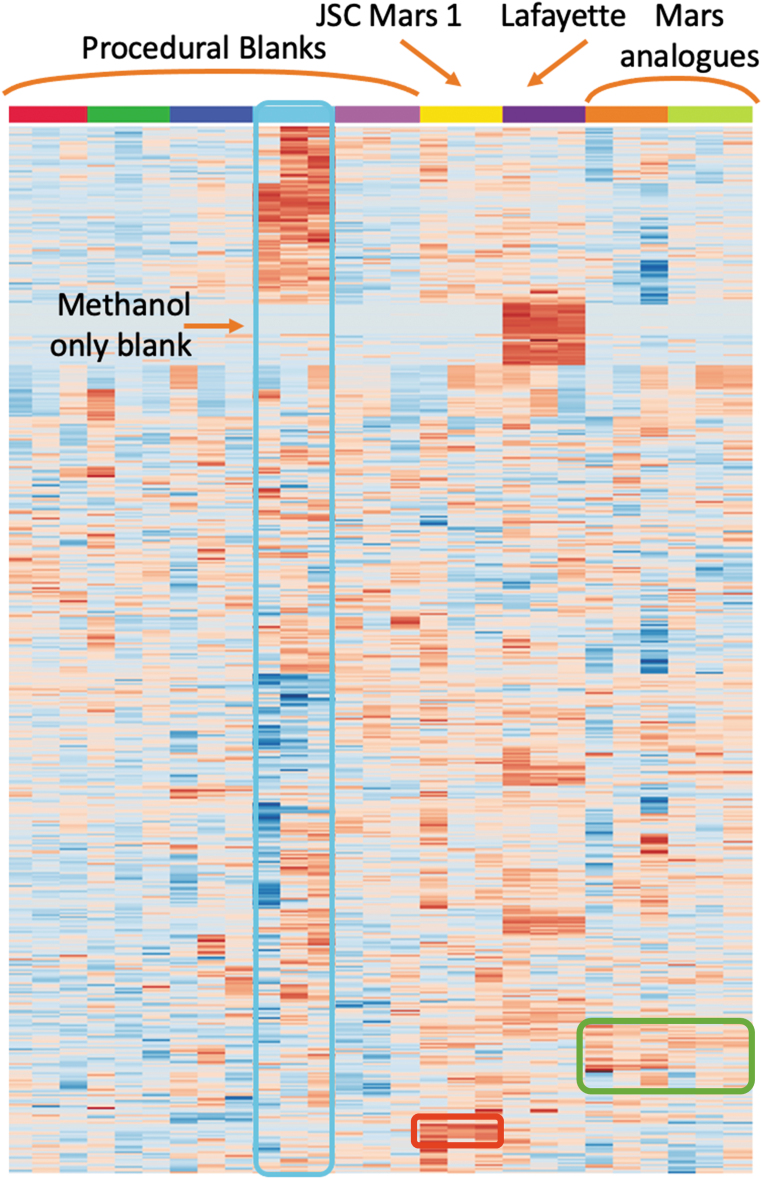
Metabolite heat map of all samples analyzed using LC-MS. Each horizontal row represents one metabolite detected; color represents relative peak intensity of that metabolite across all samples, with the deepest red representing highest peak intensity. The heat map was generated using MetaboAnalyst software. Metabolites are grouped according to similar distribution. The analog materials originate from the BVC, Svalbard, with the orange column representing the Sigurdfjellet samples and the green column the Svverefjelle samples. The heat map suggests that there are a significant number of metabolites present in the Lafayette meteorite that were below detection limits in all other samples. The heat map also suggests that there are metabolites present in JSC Mars-1 unique to those extracts (red box) and similarly for the Svalbard analogs (green box).

MetaboAnalyst was also used to generate a heat map that shows the relative levels of detected metabolites in each individual sample (Fig. 3). Here again, the Lafayette replicates are distinct, containing high relative abundances of numerous peaks that were not detected in any of the other samples or blanks. The heat map also highlights a small group of peaks that were detected in all three replicates of JSC Mars-1 in high abundance relative to the other samples (Fig. 3, red box). The two BVC Svalbard samples contain a small group of peaks that are relatively abundant within all three replicates (mostly grouped within the green box, Fig. 3). However, none of these peaks show good reproducibility across all replicates.

### 3.2. Fatty acids

**FIG. 4.**
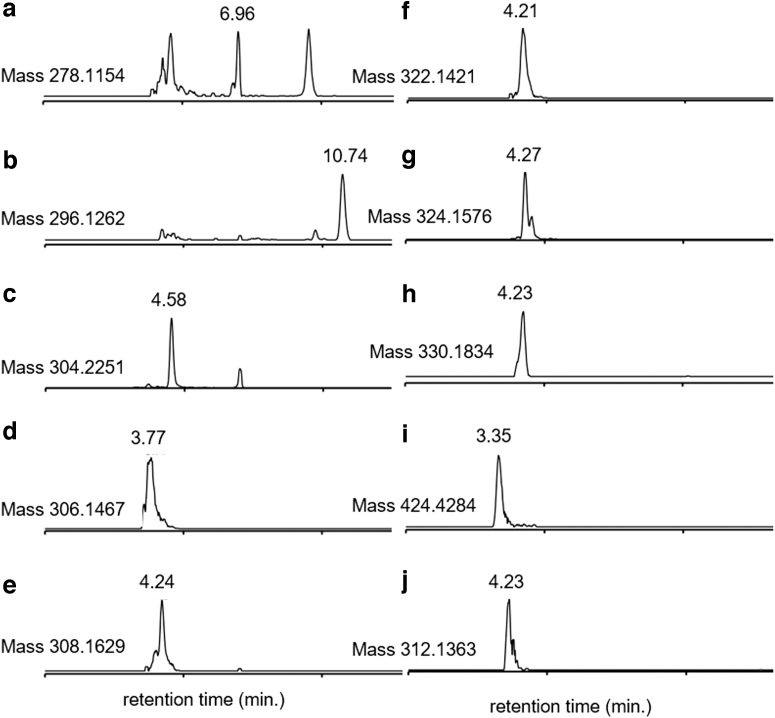
Extracted ion chromatograms of peaks of interest found via HILIC LC-MS in triplicate samples of Lafayette **(a–h)** and JSC Mars-1 **(i, j)**. The putatively annotated peaks are as follows: **(a)** artecanin/canin, **(b)** deoxynivalenol (also known as vomitoxin), **(c)** [FA trihydroxy(16:0)] 2,15,16-trihydroxy-hexadecanoic acid, **(d)** arnicolide A, **(e)** inulicin, **(f)** tetraneurin E, **(g)** tetraneurin A, and **(h)** gibberellin A15. The putatively annotated peaks in JSC Mars-1 are as follows: **(i)** octacosanoic acid and **(j)** desmosdumotin C. HILIC, hydrophilic interaction liquid chromatography.

A total of 59 of the 224 peaks detected in concentrations significantly above background in Lafayette (*i.e.*, at least twice the levels in the procedural blanks) were putatively annotated as fatty acids, including the molecule with the highest relative peak intensity [FA trihydroxy(16:0)] 2,15,16-trihydroxy-hexadecanoic acid (Fig. 4 and Supplementary Fig. SM1). Fatty acids have previously been reported in other meteorite solvent extracts, for example, via a GC-MS study of dichloromethane extractions of the CM2 chondrite Tagish Lake (Hilts *et al.,* 2014). Unlike GC-MS, this method of HILIC LC-MS is not optimized for fatty acid detection, due to the apolarity of these molecules—GC-MS would provide better separation (Schött *et al.,* 2021). Therefore, many more fatty acids may be present in Lafayette that were not detected during this study.

Nine fatty acids were also putatively annotated within JCS Mars 1, including octacosanoic acid (Fig. 4). Only one other isomer was identified for this metabolite, which is another fatty acid (6-methyl-heptacosanoic acid).

### 3.3. Terrestrial metabolites within Lafayette

Without compound-specific isotope ratios, we cannot determine with certainty whether individual metabolites detected within Lafayette have a terrestrial or martian origin. However, several metabolites were detected that are known to be produced via terrestrial biological processes and, thus, are probable terrestrial contaminants. These metabolites can help constrain the fall scenario of this meteorite. For example, six peaks with some of the highest relative abundances in the sample were putatively annotated as sesquiterpene lactones—secondary metabolites most commonly produced by plants in the Asteraceae (also known as Compositae) family. Specifically, artecanin, canin, inulicin, arnicolide A, tetraneurin A, and tetraneurin E appear to have been detected at high relative intensities in Lafayette only (Fig. 4 and Supplementary Figs. SM2–SM8).

**FIG. 5.**
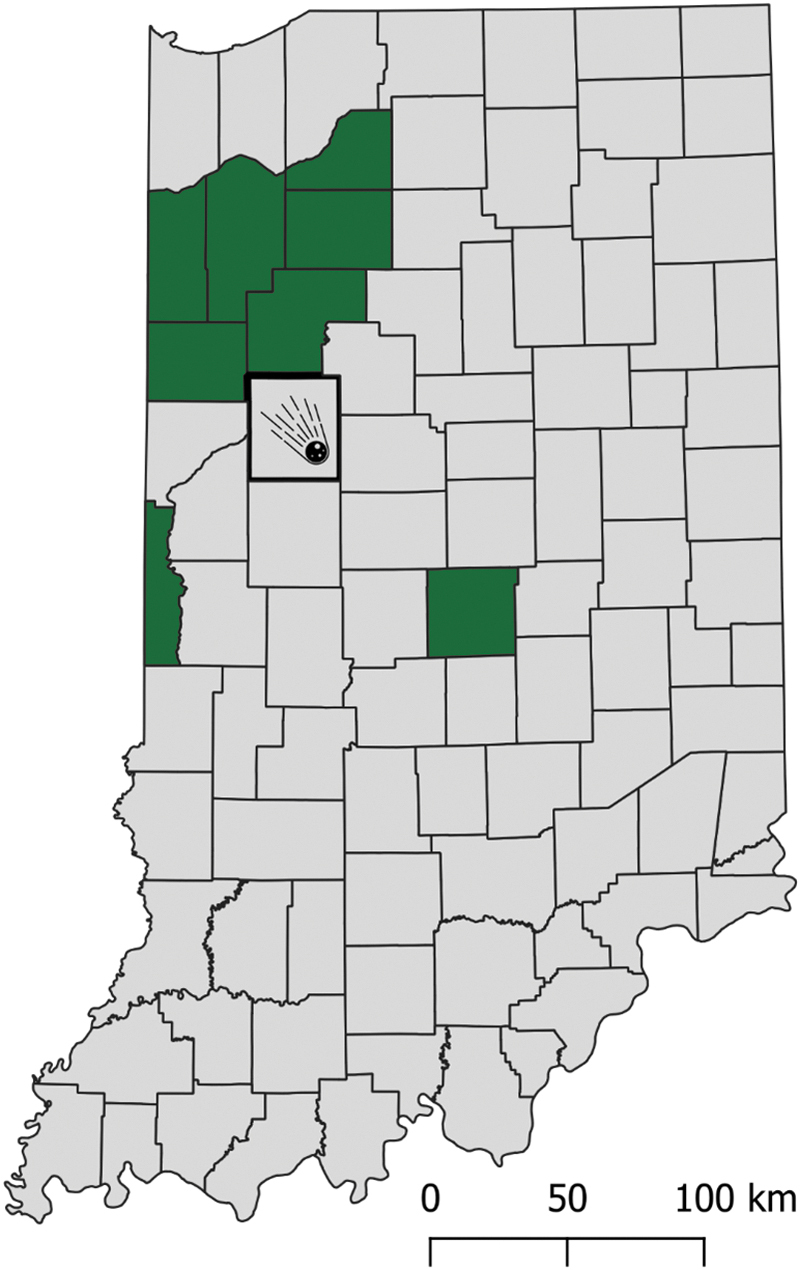
The reported fall location (Tippecanoe County) of Lafayette Meteorite compared with the native distribution of a plant whose derivative was found in the sample of Lafayette. Counties (in green) in Indiana with verified (pressed and location recorded specimens) native distribution of *Parthenium integrifolium*, the only species of *Parthenium* genus indigenous to Indiana relative to the county (in grey with a fireball symbol) where the Lafayette meteorite was reportedly first found. Plants in the *Parthenium* genus contain many sesquiterpene lactones, including those such as tetraneurin E found in this experiment and in this species. Adapted from the Indiana Plant Atlas.

Such molecules are thought to make up to 3% of the dry mass of plants in the Asteraceae family (Chadwick *et al.,* 2013), which indicates that the Lafayette stone fell into an area with such plants close by. In particular, tetraneurin E is a metabolite produced by *Parthenium*, a wildflower genus with one species *Parthenium integrifolium* (also known as wild quinine) native to the eastern United States including Indiana, where Purdue University is located (Kartesz, 2015) (Fig. 5). A standard was not run of tetraneurin E during LC-MS analysis, but no exact mass isomer for this metabolite is present in either the Human Metabolome Database (HMDB) or Kyoto Encyclopedia of Genes and Genomes databases. Therefore, it is likely that tetraneurin E is a terrestrial contaminant within Lafayette, the presence of which strengthens the case for its unconfirmed fall scenario.

Another putative metabolite detected in all three replicates of Lafayette, and in no other samples or blanks, was deoxynivalenol (DON), also known as vomitoxin (Fig. 4). This is a mycotoxin (harmful fungal chemical) released by *Fusarium graminearum*, a fungal pathogen also known as *Gibberella zeae*. This fungal pathogen causes the crop disease Fusarium head blight (FHB) of wheat and Gibberella stalk and ear rot of corn and is the dominant fungal species that causes FHB in North America (McMullen *et al.,* 2012; Munkvold and White, 2016; Schmale and Bergstrom, 2003).

There are 19 isomers (including DON) with the same *m/z* value as DON in the HMDB, and without a DON standard, we cannot be certain that DON was detected. However, the likely detection of similar plant-based metabolites, as outlined above, strengthens the case. This possibility is also reinforced by the putative detection of gibberellin A15, a fungal terpenoid with a similar molecular structure to the sesquiterpene lactones described above (Supplementary Fig. SM10). Gibberellin biosynthesis was reported in the related pathogen *Fusarium fujikuroi* (Hedden and Sponsel, 2015).

## 4. Discussion

### 4.1. OM in Lafayette versus terrestrial analogs

PCA suggests that much of the OM detected in the analogs and simulants was similar to, although distinct from, the OM of Lafayette. The metabolite detected with the highest relative intensity in Sverrefjellet, Sigurdfjellet, and JSC Mars-1 was putatively annotated as desmosdumotin C, which was below detection limits in all other samples. JSC Mars-1 and the two BVC samples were not stored together and originated from entirely different sites. However, they were stored in the same plastic sample bags, which suggests that this metabolite is a contaminant from sample storage. In contrast, the Lafayette chip was stored in aluminum foil, which perhaps explains the absence of this metabolite in the meteorite.

Many fatty acids were detected in Lafayette and JSC Mars-1 that were not detected in the procedural blanks. Interestingly, among the 25 most intense peaks in JSC Mars-1, 9 were tentatively annotated as fatty acids, which suggests that JSC Mars-1 acts as an excellent analog material for Mars in terms of its soluble organic content.

### 4.2. Constraining the fall history of Lafayette

As mentioned above, detailed knowledge surrounding the fall and recovery of Lafayette is lacking. A possible, although unconfirmed, version of events is that a Purdue University student saw the fall in Tippecanoe County (Indiana) and collected the stone from a muddy area near a pond (Nininger, 1935). Surprisingly, our LC-MS data set for Lafayette appears to support this story, particularly if we focus on the DON mycotoxin.

FHB, or “scab,” is a disease on small grains caused by the fungal pathogen *F. graminearum*. This pathogen produces the DON mycotoxin and typically causes disease on wheat in areas during favorable environmental conditions of warm temperatures and high humidity during anthesis (flowering) in the spring. FHB can cause significant yield losses and reduced grain quality. Agriculture is a major industry in Indiana and has been throughout the 19th and 20th centuries. The state's climatic and environmental conditions are ideal for *F. graminearum* to cause disease, meaning that FHB is widely recognized as a significant problem in Indiana grain. This is because the DON mycotoxin produced by *F. graminearum* is poisonous to humans in large concentrations (McMullen *et al.,* 2012). For livestock, particularly swine, it causes sickness in much lower concentrations if infected wheat is in their feed. Many pigs will refuse feed that contains ∼1 ppm of DON (Schmale and Bergstrom, 2003).

Purdue University's Department of Agronomy and Department of Botany and Plant Pathology continue to monitor and research instances of FHB in the state and best management practices for this disease (Wise and Woloshuk, 2010). The highest prevalence of the disease in Indiana within the 20 years before 1931 (when Lafayette was recognized as a meteorite) was in 1919 (Gardner, 1919). In that year, FHB caused an estimated 5–10% loss in crop yield. Detailed annual records of crop diseases at the county level within the state were kept by the Indiana National Academy of Sciences from 1919 onward. The year 1927 was also a significant year for the disease. However, the levels did not match the scale of 1919 (Gardner, 1927). Therefore, assuming the stone did fall as described in Nininger (1935), the LC-MS data presented here suggest 1919 or 1927 as the most likely fall years based on these annual disease records of FHB.

This is further supported by previous liquid chromatography studies of DON infestations in soil and crops. LC-MS/MS work by Sanders *et al.* (2013) showed that the highest concentrations of DON are found in settled infested wheat dust at five times the levels in infested grain debris. Additionally, Maiorano *et al.* (2008) used HPLC to investigate how DON concentration in soils in regions with FHB infestation varies annually, as well as with mitigative strategies such as crop tilling. They found that minimum DON concentrations varied between a minimum of 125 μg kg^−1^ in their 2004 samples, a minimum of 25 μg kg^−1^ in their 2005 samples, to a minimum DON concentration of 1192 μg kg^−1^ in their 2006 samples.

In line with this, 2006 had a much higher FHB infestation in the wheat crops studied, and other mitigation efforts (such as tilling) had a negligible impact on DON concentration. Maiorano *et al.* thus hypothesized that annual infestation has the biggest impact on DON concentration in their soil samples. Given that Nininger's account of the fall gives the stone as falling in the mud at the edge of a pond, we suggest that it is highly likely to have been near a field infested with FHB at the time, with a layer of infested dust at its surface, and it was likely to have been in a year of very high FHB infestation to be detected so many years later.

The student who saw the fall is reported to have been a student of color registered at Purdue University before 1931 (Nininger, 1935). During this period at Purdue, Black students were referred to as students of color, while students of other non-White ethnicities were referred to as foreign students. By the time the stone was identified as a meteorite, the student's name was forgotten, so the fall scenario remained unconfirmed (Nininger, 1935). Very few students of color attended Purdue University in the late 1910s, to the point that there were no Black students known to have graduated in 1919 or 1920. However, there were three Black students known to have been in the university in 1919—two pharmaceutical students in the graduating class of 1921, and one engineering student in the graduating class of 1922. There was only one Black student known to have attended Purdue in 1927. Therefore, both years fit the reported fall scenario; however, the higher prevalence of FHB in 1919 makes this year the most probable.

Intriguingly, there were also well-documented fireballs in the Midwestern USA in both 1919 and 1927. A fireball event in 1927 is known to have dropped the Tilden meteorite in Illinois, a chondrite meteorite. On November 26, 1919, at 8 pm, a fireball was observed primarily in Southern Michigan and northern Indiana, with additional reported sightings across the Midwest and into Canada (Hobbs, 1923). Hobbs (1923) suggested that, due to the length of the fireball, the shockwave was felt around Lake Michigan and into Canada, and the clear multiple paths were observed as well such that a stone was likely to have fallen onto land east of Lake Michigan and near the town of Portage, Michigan.

A couple of searches took place, but a meteorite was never found. However, due to extensive cloud cover in most of Michigan, the actual trajectory and fall position are unclear, although it is unlikely that this would account for the 220 km distance between the estimated fall site of Portage and Lafayette. The accounts of the Lafayette fall scenario imply daylight at the time, possibly explaining the absence of other fireball reports for this fall.

### 4.3. Implications for MSR and curation

The detection of multiple terrestrial plant-derived secondary metabolites within an interior chip of Lafayette illustrates how easily terrestrial OM can contaminate martian samples. Despite the uncertainty surrounding its fall history, Lafayette's lack of terrestrial weathering features and black glassy fusion crust attest to its relatively pristine nature (Nininger, 1935). The persistence of these plant-derived contaminants decades after the stone fell, and the fact that they are among the most abundant metabolites detected, indicates that biogenic OM ingress into extraterrestrial samples can easily and rapidly occur throughout an entire meteorite mass and can be preserved for many years.

MSR aims to bring back soil and rock samples from the martian surface for further study here on Earth. One of the key aims of these Earth-based studies will be to catalog the martian OM present within these samples (IMOST, 2018). LC-MS metabolomics as described here could help achieve this, with the optimal work plan including initial nontargeted analyses followed by targeted analyses alongside standards to identify specific molecular isomers (Seyler *et al.,* 2020). However, considering that martian OM could contain some molecules with the same structure as potential terrestrial contaminants, the only way to identify the true source of these molecules would be compound-specific isotope ratio analyses, currently only available via gas chromatography isotope ratio mass spectrometry. However, it has been suggested that the current detection limits of this technique require sample sizes of ∼1 kg (Callahan *et al.,* 2014), which is unfeasible for such precious samples.

The small sample sizes utilized for this study (3 × 30 mg replicates) coupled with the large number of metabolites detected—some of which are almost certainly plant-derived material from ∼100 years ago—suggest that similar LC-MS protocols would be valuable during the investigation of OM in MSR.

## 5. Conclusions

The aim of this study was to evaluate a nontargeted metabolomics approach to organics detection in martian meteorites, both to characterize contamination and to identify any possible indigenous (*i.e.*, martian) OM. In this study, we show that room temperature solvent extractions with the use of hexane, dichloromethane, and methanol—complete with a comprehensive contamination control using blanks—capture a broad range of metabolites that can be detected by LC-MS. When combined with rigorous bioinformatics analysis, this technique is effective as a nontargeted approach to metabolomics, and a follow-up targeted approach with standards would allow for the confirmation of metabolites detected.

Although Lafayette appears to be a pristine martian sample, our nontargeted metabolomics study suggests that there are a number of terrestrial plant-derived organic contaminants within the meteorite. The presence of these contaminants emphasizes the need for carefully controlled and documented curation of martian returned samples to ensure that terrestrial organic contamination does not take place.

The putative identification of vomitoxin (DON) and other plant-derived metabolites within Lafayette appears to support the unconfirmed report that the meteorite fell in the Indiana countryside, as outlined by Nininger (1935). The putative detection of vomitoxin, a mycotoxin produced by the fungal pathogen *F. graminearum*, suggests the stone fell during a year when FHB was most severe. Archives relating to FHB from the Indiana Academy of Sciences suggest 1919 as the most severe blight year during the possible fall window of 62 years. This year also corresponds with the unconfirmed report that the student who saw the fall and collected the meteorite was a student of color, based on Purdue University's yearbook and graduation records.

This work demonstrates the importance of studying terrestrial contaminants in meteorites, particularly for rare astrobiologically important stones with unconfirmed fall histories. The LC-MS data presented here, combined with archival and historical “detective work,” have elucidated our knowledge of Lafayette's terrestrial history.

## Supplementary Material

Supplemental data

Supplemental data

## Supplementary Material

Supplementary Materials SM1

Supplementary Materials SM2

## Abbreviations Used

BVC: Bockfjorden volcanic complex
DCM: dichloromethane
DON: deoxynivalenol
FHB: Fusarium head blight
GC-MS: gas chromatography–mass spectrometry
HILIC-MS: hydrophilic interaction liquid chromatography–mass spectrometry
HMDB: Human Metabolome Database
HPLC: high-performance liquid chromatography
LC-MS: liquid chromatography–mass spectrometry
MMC: macromolecular carbon
MSR: Mars Sample Return
OM: organic matter
PCA: principal component analysis
PTFE: polytetrafluoroethylene
SUERC: Scottish Universities Environmental Research Centre

## Notes

Associate Editor: Nita Sahai

## References

[B1] Allen CC, Morris RV, Karen MJ, et al. Martian regolith simulant JSC Mars-1. In 29th Annual Lunar and Planetary Science Conference, March 16-20, 1998, Houston, TX, abstract no. 1690, https://www.lpi.usra.edu/meetings/LPSC98/pdf/1690.pdf.

[B2] Amundsen HEF, Benning L, Blake DF, et al. Cryogenic origin for Mars analog carbonates in the Bockfjord volcanic complex, Svalbard (Norway). In: 42nd Lunar and Planetary Science Conference (No. JSC-CN-22844), March 7, 2011, Woodlands, TX, 20110008112.pdf

[B3] Becker L, Popp B, Rust T, et al. The origin of organic matter in the martian meteorite ALH84001. Earth Planet Sci Lett 1999;167(1–2):71–79; doi: 10.1016/S0012-821X(99)00014-X 11542930

[B4] Biemann K, Oro J, Toulmin III P, et al. The search for organic substances and inorganic volatile compounds in the surface of Mars. J Geophys Res 1977;82(28):4641–4658; doi: 10.1029/js082i028p04641

[B5] Borg L, Drake MJ. A review of meteorite evidence for the timing of magmatism and of surface or near-surface liquid water on Mars. J Geophys Res 2005;110(12):1–10; doi: 10.1029/2005JE002402

[B6] Bunch TE, Reid AM. The nakhlites Part I: Petrography and mineral chemistry. Meteoritics 1975;10:303–315; doi:10.1111/j.1945-5100.1975.tb01187.x

[B7] Callahan MP, Burton AS, Elsila JE, et al. A search for amino acids and nucleobases in the martian meteorite Roberts Massif 04262 using liquid chromatography-mass spectrometry. Meteorit Planet Sci 2013;48(5):786–795; doi: 10.1111/maps.12103

[B8] Callahan MP, Martin MG, Burton AS, et al. Amino acid analysis in micrograms of meteorite sample by nanoliquid chromatography-high-resolution mass spectrometry. J Chromatogr A 2014;1332:30–34; doi: 10.1016/j.chroma.2014.01.032 24529954

[B9] Chadwick M, Trewin H, Gawthrop F, et al. Sesquiterpenoids lactones: Benefits to plants and people. Int J Mol Sci 2013;14(6):12780–12805; doi: 10.3390/ijms140612780 23783276 PMC3709812

[B10] Cohen BE, Mark DF, Cassata WS, et al. Taking the pulse of Mars via dating of a plume-fed volcano. Nat Commun 2017;8(1):1–8; doi: 10.1038/s41467-017-00513-8 28974682 PMC5626741

[B11] Creek DJ, Andris Jankevics Karl EV, Burgess, et al. IDEOM: an Excel interface for analysis of LC–MS-based metabolomics data. Bioinformatics 2012;28(7)1048–1049; doi: 10.1093/bioinformatics/bts069 22308147

[B12] Daly L, Lee MR, Piazolo S, et al. Boom boom pow: Shock-facilitated aqueous alteration and evidence for two shock events in the Martian nakhlite meteorites. Science Advances 2019;5(9); doi: 10.1126/sciadv.aaw5549 PMC6726442 31517047

[B13] Eigenbrode JL, Summons RE, Steele A, et al. Organic matter preserved in 3-billion-year-old mudstones at Gale crater, Mars. Science 2018;360(6393):1096–1101; doi: 10.1126/science.aas9185 29880683

[B14] Forcisi S, Moritz F, Kanawati B, et al. Liquid chromatography-mass spectrometry in metabolomics research: Mass analyzers in ultra high pressure liquid chromatography coupling. J Chromatogr A 2013;1292:51–65; doi: 10.1016/j.chroma.2013.04.017 23631876

[B15] Gardner MW. Indiana plant diseases, In Proceedings of the Indiana Academy of Science 1919, Vol. 29, pp. 135–156.

[B16] Gardner MW. Indiana plant diseases, In Proceedings of the Indiana Academy of Science 1927, Vol. 38, pp. 143–158.

[B17] Glavin DP, Aubrey AD, Callahan MP, et al. Extraterrestrial amino acids in the Almahata Sitta meteorite. Meteorit Planet Sci 2010;45:1695–1709.

[B18] Glavin DP, Bada JL, Brinton KLF, et al. Amino acids in the martian meteorite Nakhla. Proc Natl Acad Sci U S A 1999;96(16):8835–8838; doi: 10.1073/pnas.96.16.8835 10430856 PMC17693

[B19] Glavin DP, Dworkin JP, Aubrey A, et al. Amino acid analyses of Antarctic CM2 meteorites using liquid chromatography-time of flight-mass spectrometry. Meteorit Planet Sci 2006;41:889–890.

[B20] Gloaguen Y, Morton F, Daly R, et al. PiMP my metabolome: an integrated, web-based tool for LC-MS metabolomics data. Bioinformatics 2017;33(24):4007–4009. 28961954 10.1093/bioinformatics/btx499 PMC5860087

[B21] Guzman M, Szopa C, Freissinet C, et al. Testing the capabilities of the Mars organic molecule analyser (MOMA) chromatographic columns for the separation of organic compounds on Mars. Planet Space Sci 2020;186:104903; doi: 10.1016/j.pss.2020.104903

[B22] Hedden P, Sponsel V. A century of Gibberellin research. J Plant Growth Regul 2015;740–760; doi: 10.1007/s00344-015-9546-1 26523085 PMC4622167

[B23] Hilts RW, Herd CDK, Simkus DN, et al. Soluble organic compounds in the Tagish Lake meteorite. Meteorit Planet Sci 2014;49(4):526–549; doi: 10.1111/maps.12272

[B24] Hobbs WH. The Southwestern Michigan Meteor of November 26, 1919. In: Papers of the Michigan Academy of Science, Arts, and Letters 1923;1:253–268.

[B25] IMOST. The potential science and engineering value of samples delivered to Earth by Mars sample return. (co-chairs D. W. Beaty, M. M. Grady, H. Y. McSween, E. Sefton-Nash; documentarian B.L. Carrier; plus 66 co-authors). White Paper, 2018; p. 186.

[B26] Jankevics A, Merlo ME, de Vries M, et al. Separating the wheat from the chaff: a prioritisation pipeline for the analysis of metabolomics datasets. Metabolomics 2012;8(1):29–36. 22593722 10.1007/s11306-011-0341-0 PMC3337394

[B27] Kvenvolden KA, Lawless JG, Ponnamperuma C. Nonprotein Amino Acids in the Murchison Meteorite. In Proceedings of the National Academy of Sciences 1971;68(2). 10.1073/pnas.68.2.486 PMC388966 16591908

[B28] Lin Y, El Goresy A, Hu S, et al. NanoSIMS analysis of organic carbon from the tissint martian meteorite: Evidence for the past existence of subsurface organic-bearing fluids on Mars. Meteorit Planet Sci 2014;49(12):2201–2218; doi: 10.1111/maps.12389

[B29] Maiorano A, Blandino M, Reyneri A, et al. Effects of maize residues on the Fusarium spp. infection and deoxynivalenol (DON) contamination of wheat grain. Crop Prot 2008;27:182–188.

[B30] Mari N, Riches AJV, Hallis LJ, et al. Syneruptive incorporation of martian surface sulphur in the nakhlite lava flows revealed by S and Os isotopes and highly siderophile elements: Implication for mantle sources in Mars. Geochim Cosmochim Acta 2019;266:416–434.

[B31] McMullen M, Bergstrom G, De Wolf E, et al. A unified effort to fight an enemy of wheat and barley: Fusarium head blight. Plant Disease 2012;96(12);1712–1728. 30727259 10.1094/PDIS-03-12-0291-FE

[B32] Morris RV, Golden DC, Bell JF 3rd, et al. Pigmenting agents in martian soils: Inferences from spectral, Mössbauer, and magnetic properties of nanophase and other iron oxides in Hawaiian palagonitic soil PN-9. Geochim Cosmochim Acta 1993;57(19):4597–4609; doi: 10.1016/0016-7037(93)90185-Y 11539577

[B33] Munkvold, Gary Phillip, Donald G. White, eds. Compendium of corn diseases. Vol. 165. St. Paul, Minnesota, USA: APS Press, 2016.

[B34] Nininger H. The Lafayette meteorite. Popular Astron 1935;XLIII:404–408.

[B35] Rull F, Maurice S, Hutchinson I, et al. The Raman laser spectrometer for the ExoMars rover mission to Mars. Astrobiology 2017;17(6–7):627–654; doi: 10.1089/ast.2016.1567

[B36] Sagan C, Lederberg J. The prospects for life on Mars: A pre-viking assessment. Icarus 1976;28(2):291–300; doi: 10.1016/0019-1035(76)90039-7

[B37] Sanders M, De Boevre M, Dumoulin F, et al. Sampling of wheat dust and subsequent analysis of deoxynivalenol by LC-MS/MS. J Agric Food Chem 2013;61(26):6259–6264; doi: 10.1021/jf401323s 23782015

[B38] Schött HF, Konings MCJM, Schrauwen-Hinderling VB, et al. A validated method for quantification of fatty acids incorporated in human plasma phospholipids by gas chromatography-triple quadrupole mass spectrometry. ACS Omega 2021;6(2):1129–1137; doi: 10.1021/acsomega.0c03874 33490772 PMC7818123

[B39] Sephton MA, Wright IP, Gilmour I, et al. High molecular weight organic matter in martian meteorites. Planet Space Sci 2002;50(7–8):711–716; doi: 10.1016/S0032-0633(02)00053-3

[B40] Seyler L, Kujawinski EB, Azua-Bustos A, et al. Metabolomics as an emerging tool in the search for astrobiologically relevant biomarkers. Astrobiology 2020;20(10):1251–1261; doi: 10.1089/ast.2019.2135 32551936 PMC7116171

[B41] Siljeström S, Freissinet C, Goesmann F, et al. Comparison of prototype and laboratory experiments on MOMA GCMS: Results from the AMASE11 campaign. Astrobiology 2014;14(9):780–797; doi: 10.1089/ast.2014.1197 25238325

[B42] Simkus DN, Aponte JC, Elsila JE, et al. Methodologies for analyzing soluble organic compounds in extraterrestrial samples: Amino Acids, amines, monocarboxylic acids, aldehydes, and ketones. Life 2019;9(2); doi: 10.3390/life9020047 PMC6617175 31174308

[B43] Smith CA, Want EJ, O'Maille G, et al. XCMS: processing mass spectrometry data for metabolite profiling using nonlinear peak alignment, matching, and identification. Anal Chem 2006;78(3):779–787. 16448051 10.1021/ac051437y

[B44] Steele A, Benning L, Wirth R, et al. Organic synthesis on Mars by electrochemical reduction of CO_2_. Sci Adv 2018;4(10):eaat5118; doi: 10.1126/sciadv.aat5118 30402538 PMC6209388

[B45] Steele A, Fries MD, Amundsen HEF, et al. Comprehensive imaging and Raman spectroscopy of carbonate globules from martian meteorite ALH 84001 and a terrestrial analogue from Svalbard. Meteorit Planet Sci 2007;42(9):1549–1566; doi: 10.1111/j.1945-5100.2007.tb00590.x

[B46] Steele A, McCubbin FM, Fries M, et al. A reduced organic carbon component in martian basalts. Science 2012;337(6091):212–215; doi: 10.1126/science.1220715 22628557

[B47] Steele A, McCubbin FM, Fries MD. The provenance, formation, and implications of reduced carbon phases in martian meteorites. Meteorit Planet Sci 2016;51(11):2203–2225; doi: 10.1111/maps.12670

[B48] Toporski J, Steele A. Observations from a 4-year contamination study of a sample depth profile through the martian meteorite Nakhla. Astrobiology 2007;7(2):389–401; doi: 10.1089/ast.2006.0009 17480167

[B49] Treiman AH. The nakhlite meteorites: Augite-rich igneous rocks from Mars. Chem Erde 2005;65(3):203–270; doi: 10.1016/j.chemer.2005.01.004

[B50] Udry A, Howarth GH, Herd CDK, et al. What martian meteorites reveal about the interior and surface of Mars. J Geophys Res Planets 2020;125(12):1–34; doi: 10.1029/2020JE006523

[B51] Vago JL, Westall F, Coates AJ, et al. Habitability on Early Mars and the Search for Biosignatures with the ExoMars Rover. Astrobiology 2017;17(6–7):471–510; doi: 10.1089/ast.2016.1533 31067287 PMC5685153

[B52] Webster CR, Mahaffy PR, Atreya SK, et al. Mars methane detection and variability at Gale crater. Science 2015;347(6220):415–417; doi: 10.1126/science.1261713 25515120

[B53] Wise K, Woloshuk C, Freije A. Diseases of wheat: Fusarium head blight (head scab). Purdue Extension 2010;1–4.

[B54] Xia J, Psychogios N, Young N, Wishart DS. MetaboAnalyst: a web server for metabolomic data analysis and interpretation. Nucleic Acids Re 2009;37(suppl_2):W652–W660. 10.1093/nar/gkp356 PMC2703878 19429898

