# Supplementary material for: Using Organic Contaminants to Constrain the Terrestrial Journey of the Martian Meteorite Lafayette
Source: Astrobiology. 2022 Oct 31;22(11):1351–62. doi: 10.1089/ast.2021.0180 (PMC9618387; doi:10.1089/ast.2021.0180)
Supplement: Supplemental data [file Suppl_MaterialSM1.docx]

**Using Organic Contaminants to Constrain the Terrestrial Journey of the Martian Meteorite Lafayette**

**– SUPPLEMENTARY MATERIALS Part 1**

**Explanation of IDEOM Supplementary file**

The ‘Comparison’ sheet contains a list of putatively annotated metabolites in the samples. Columns A and B contain the exact neutral mass (in Da) and retention time (in min), respectively. Column C shows the formula that has been assigned to a feature by matching its detected m/z to m/z in the IDEOM database (a 3 ppm window for mass identification was applied). Column D contains the number of chemical isomers in the database sharing the formula in column C. Column E shows the metabolite name (from the database) which best matches the exact mass and retention time for the peak based on the retention time prediction algorithm. Clicking on the drop-down menu enables to visualise the names of all other isomers in the database. Metabolites highlighted in yellow are the ones which were matched to an authentic standard (a retention time window of 5% was applied). Column F contains an indicative confidence score (0-10) regarding metabolite annotation based on the parameters in the ‘settings’ sheet is given in column F. Columns G and H show the general area of metabolism and a list of biochemical pathways that the putative metabolite is an intermediate of. Column I shows the highest intensity obtained for each peak across all samples. Columns J-S shows the fold-change difference in mean peak intensity between each sample group and the FBD1 group. Columns T-AC show the P-values calculated for unpaired Student’s t-test between each sample group and the FBD1 group. Columns AD-AM show the mean peak intensity values for the three study groups. The standard deviation and relative standard deviation (RSD) for each group can be found in columns AN-AW and AX-BG respectively. Column BH-BQ show Fisher’s ratios each sample group and the FBD1 group. Column BR shows the unique identifier (PeakID) that was assigned to each peak. The ‘alldata’ sheet contains additional information on all detected peaks (with the corresponding PeakID in column BT), including relative intensities in individual samples (columns AB-BN). Additional information on the other sheets in the IDEOM dataset and on data processing using IDEOM can be found in (Creek *et al.* 2012).

**Additional information on metabolites of Interest**

| 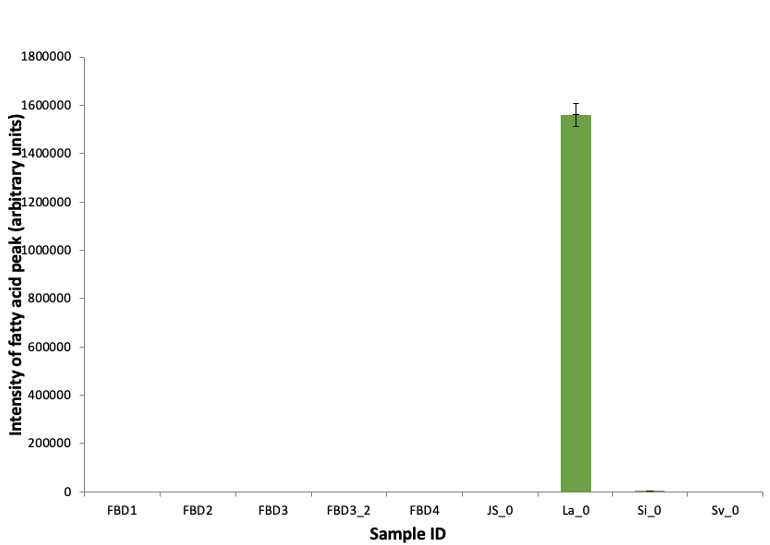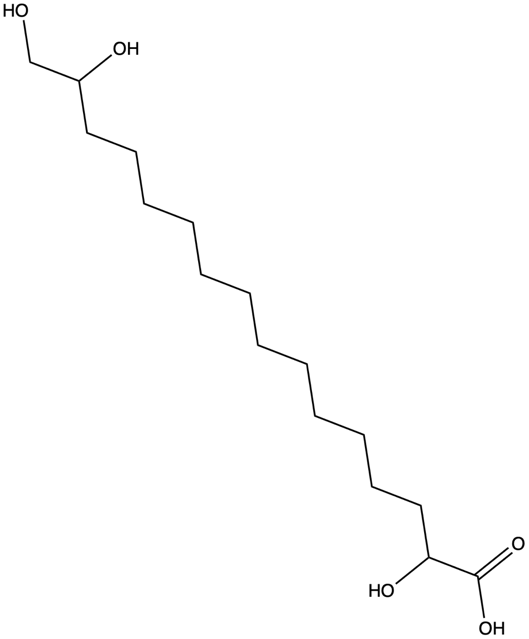 Figure SM1 Peak intensity distribution of metabolite putatively identified as [FA trihydroxy(16:0)] 2,15,16-trihydroxy-hexadecanoic acid. This fatty acid was detected in both positive and negative modes, with a retention time of 4.5808 minutes and an exact mass of 304.2251. The structure of the molecule is also shown. |
| --- |
| Figure SM2 LCMS peak intensity distribution of metabolite putatively annotated as octacosanoic acid, a fatty acid. The metabolite was detected in negative mode with a retention time of 3.350 minutes corresponding to an exact mass of 424.428417. The structure of octacosanoic acid is also shown. 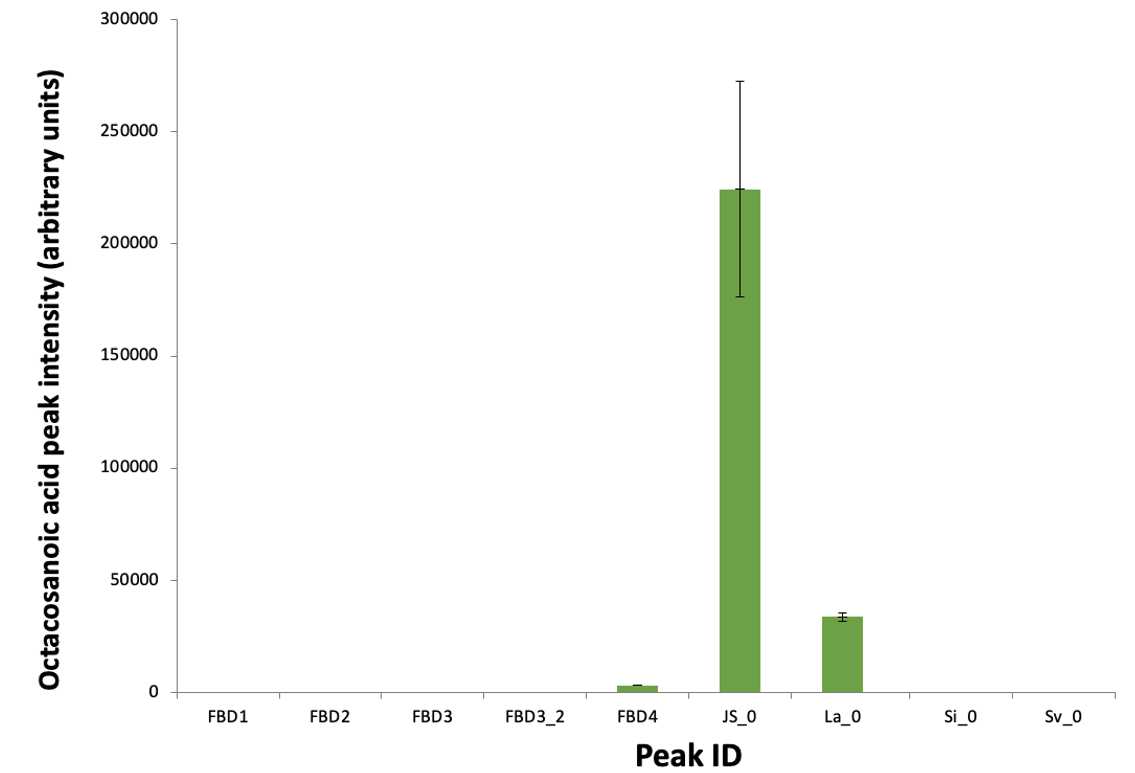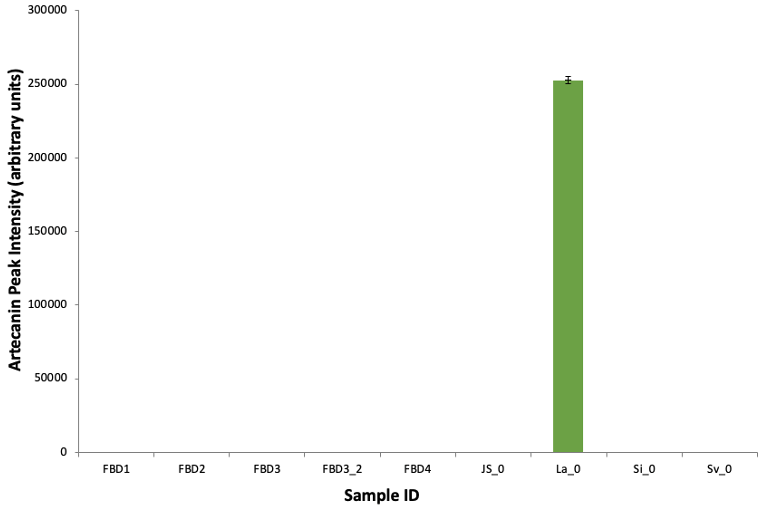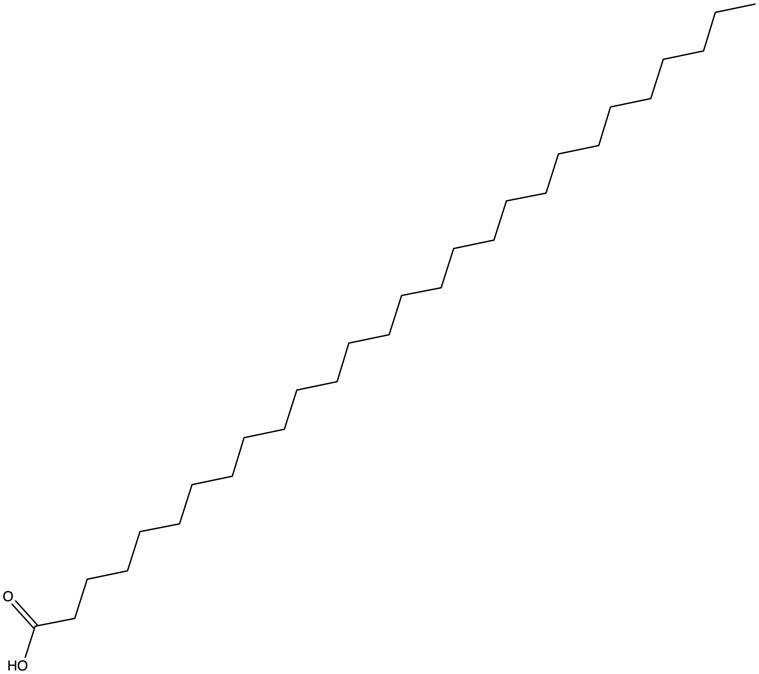 |
| 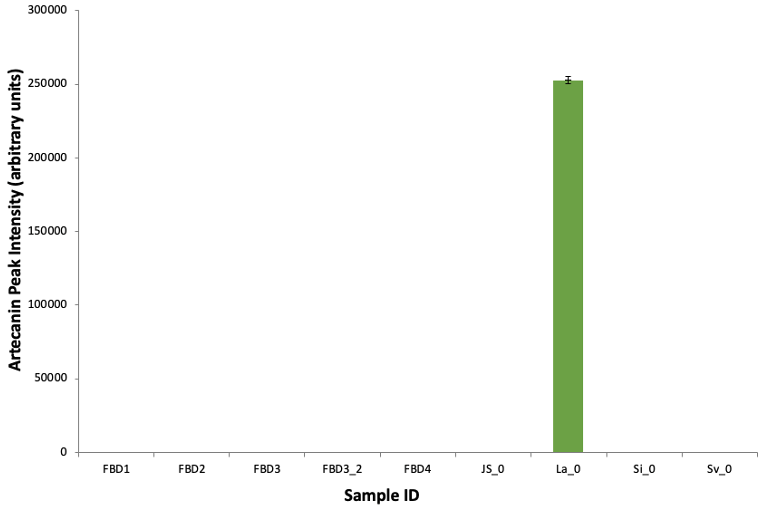 Figure SM3 LCMS peak intensity distribution of metabolite putatively annotated as artecanin. The metabolite was detected in negative mode with a retention time of 6.96249 minutes corresponding to an exact mass of 278.11543. Artecanin and its stereoisomer canin (see Figure SM4) are sesquiterpene lactones, metabolites similar to tetraneurin A and E, inlulicin, and arnicolide A below, strengthening the case for the identification of this likely terrestrial contaminant. The structure of artecanin is also shown. 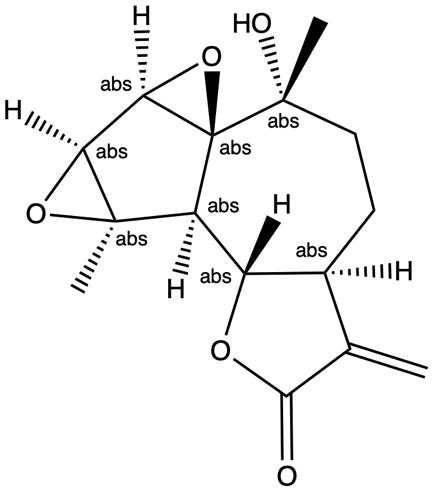 |
| Figure SM4 LCMS peak intensity distribution of metabolite putatively annotated as canin. The metabolite was detected in negative mode with a retention time of 9.562367 minutes corresponding to an exact mass of 278.1154777. No standard of canin was run, and there are 11 isomers with the same m/z value. Canin and its stereoisomer artecanin (see Figure SM3) are sesquiterpene lactones, metabolites similar to tetraneurin A and E below, strengthening the case for the identification of this likely terrestrial contaminant. The structure of canin is also shown. 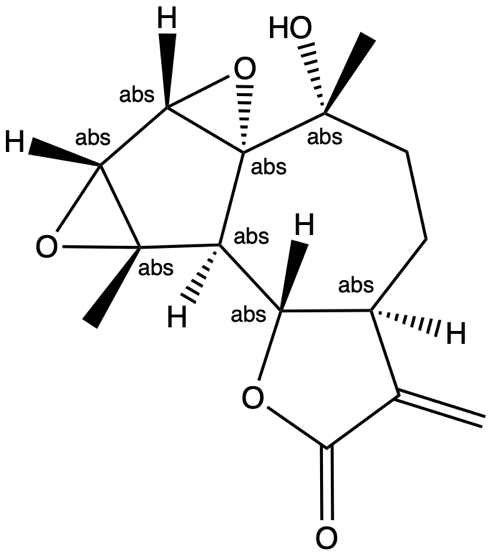 |
| 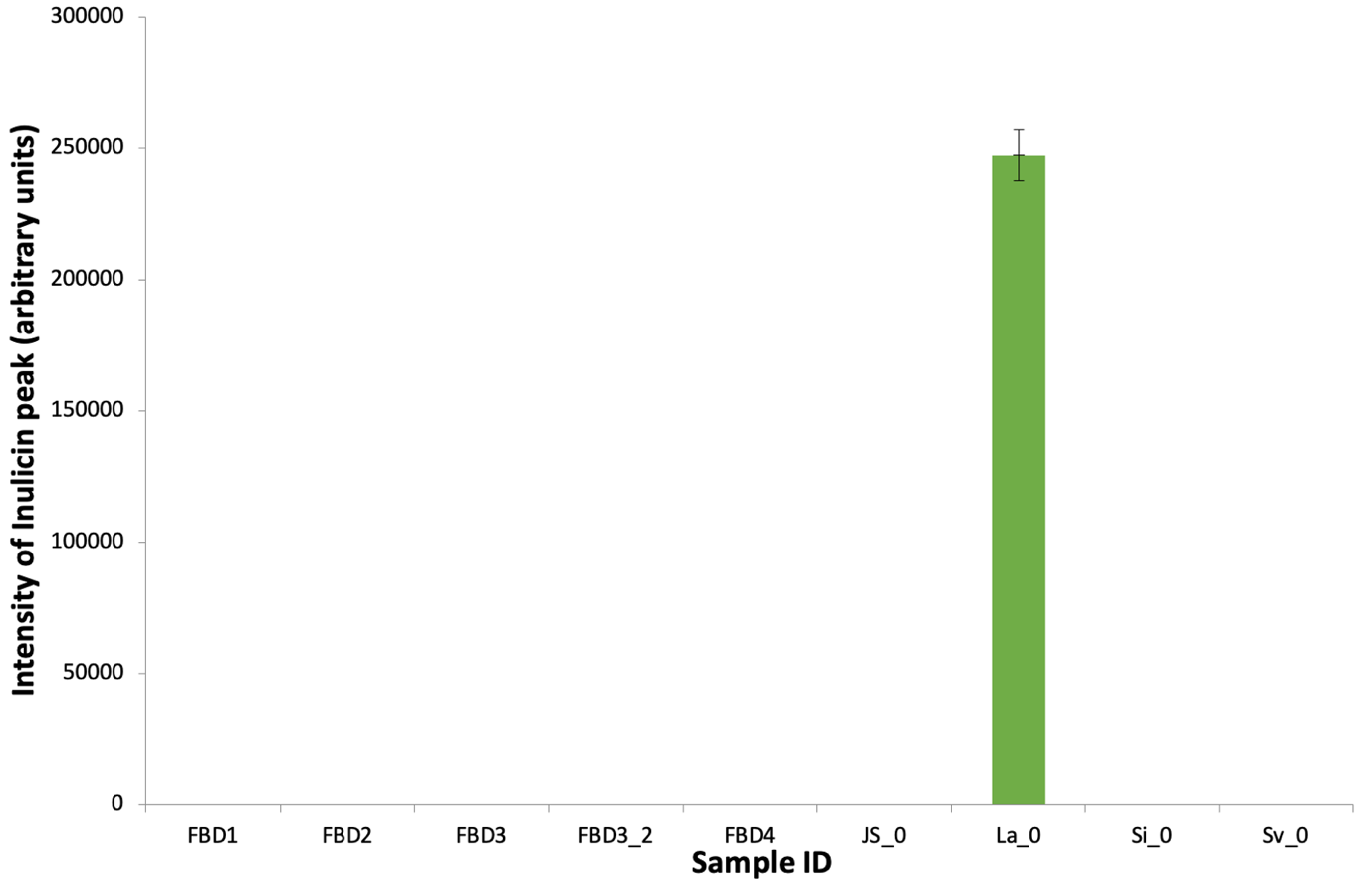 Figure SM5 LCMS peak intensity distribution of metabolite putatively annotated as Inulicin. The metabolite was detected in negative mode with a retention time of 4.2436 minutes corresponding to an exact mass of 308.1629. No standard of Inulicin was run. Inulicin is a sesquiterpene lactone, similar to those above and below, strengthening the case for the identification of this likely terrestrial contaminant. The structure of Inulicin is also shown. 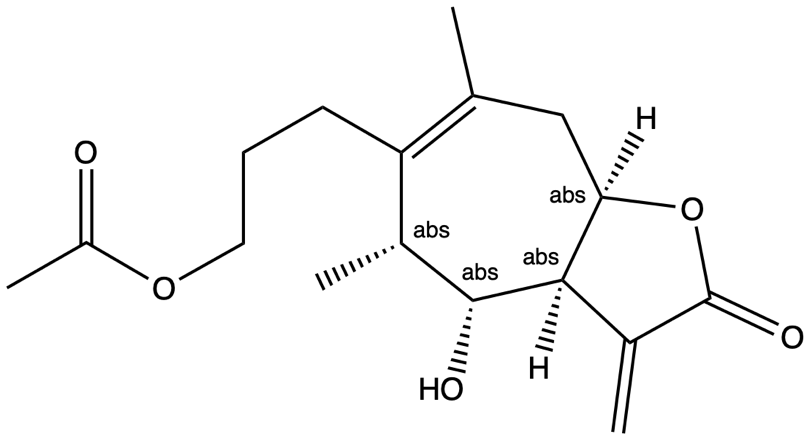 |
| 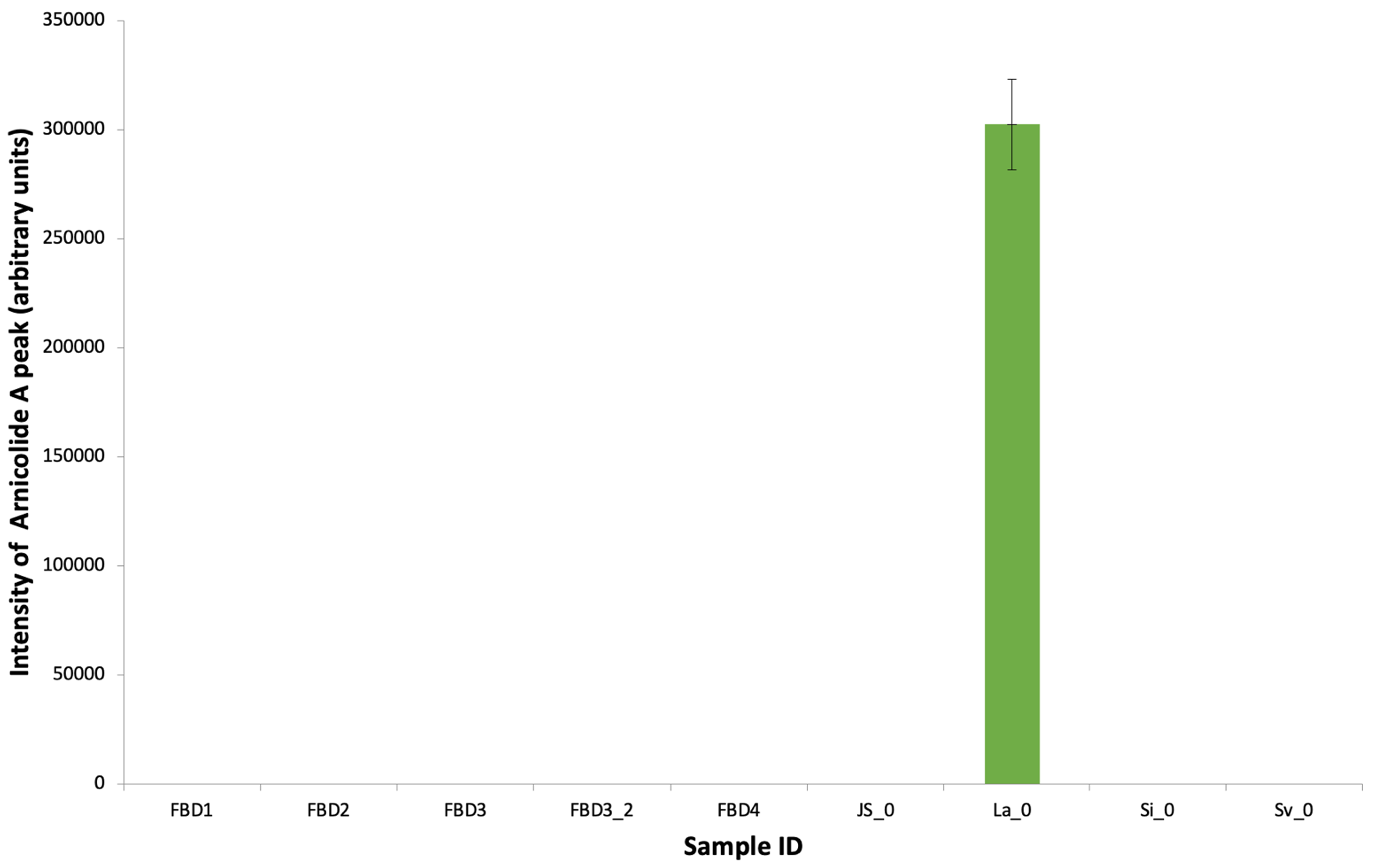 Figure SM6 LCMS peak intensity distribution of metabolite putatively annotated as Arnicolide A. The metabolite was detected in both negative and positive modes with a retention time of 3.774 minutes corresponding to an exact mass of 306.1467. No standard of arnicolide A was run. Arnicolide A is a sesquiterpene lactone, like those above and below, strengthening the case for the identification of this likely terrestrial contaminant. The structure of Arnicolide A is also shown. 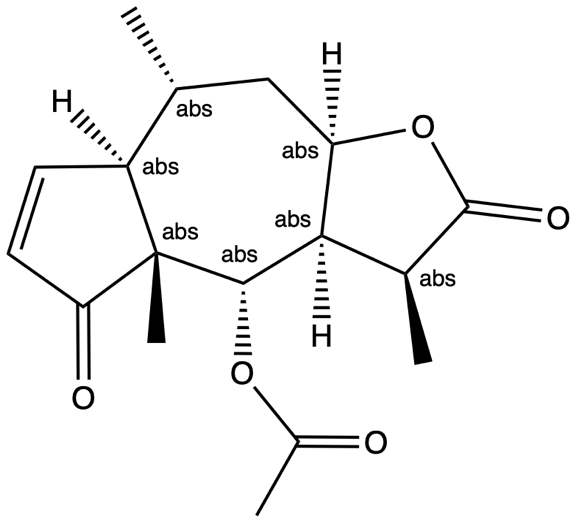 |
| 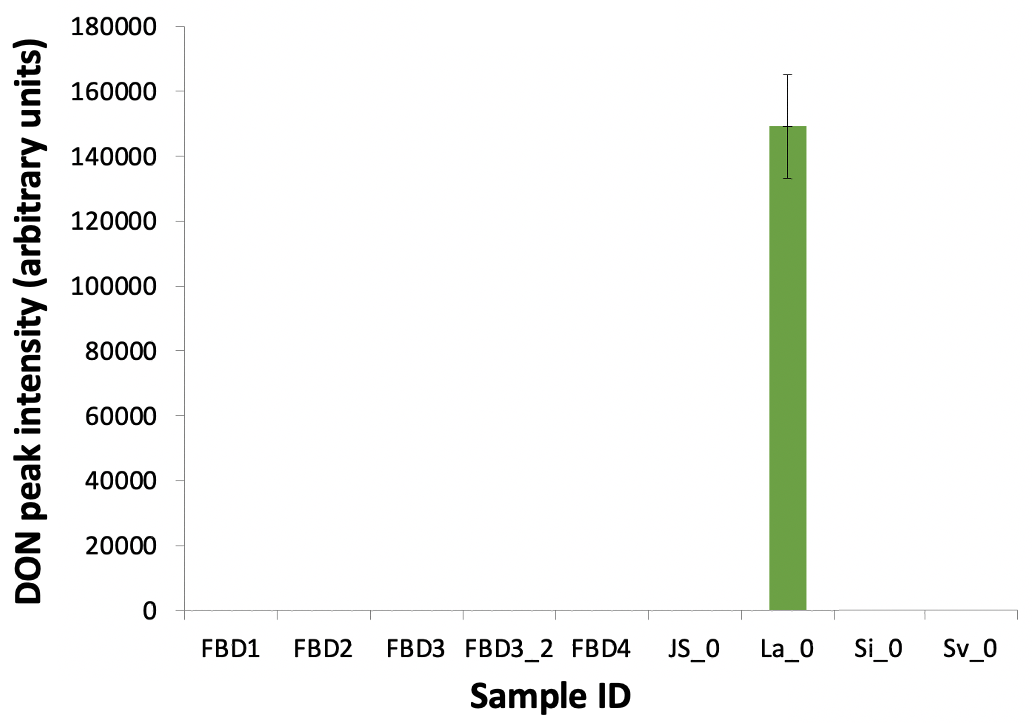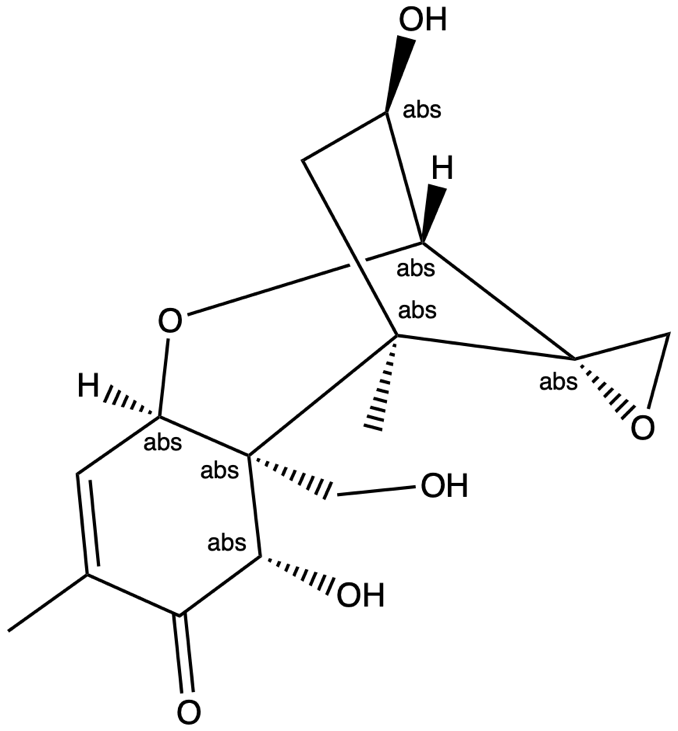 Figure SM7 LCMS Peak intensity distribution of metabolite found in negative mode that was putatively annotated as Tetraneurin A. This molecule had a retention time of 4.2142 minutes and exact mass of 322.14207. Only one other isomer with the same m/z value was found in the KEGG database. Tetraneurin A is a sesquiterpene lactone similar to those above and below. The structure of Tetraneurin A is also shown. |
| 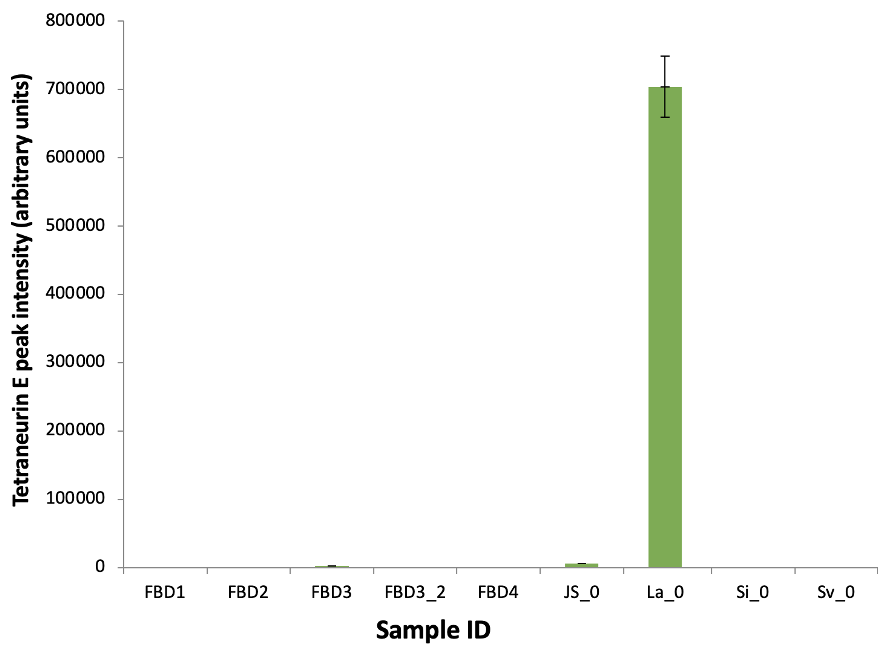 Figure SM8 LCMS Peak intensity distribution of metabolite found in negative mode that was putatively annotated as Tetraneurin E (also known as ambrosanolide). This molecule had a retention time of 4.267 minutes and exact mass of 324.1576. It was found to have the second highest peak intensity in Lafayette relative to the procedural blanks, amongst all metabolites detected in the samples. Tetraneurin E is a sesquiterpene lactone similar to those above. The structure of Tetraneurin E is also shown. 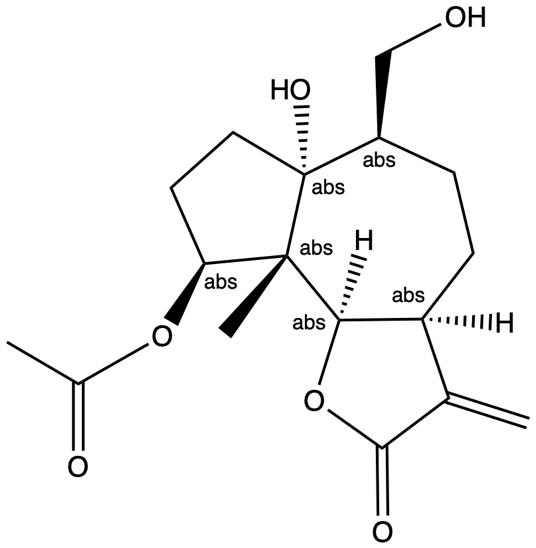 |
| Figure SM 9 LCMS Peak intensity distribution of metabolite found in negative mode that was putatively identified as deoxynivalenol (DON), also known as vomitoxin. This molecule had a retention time of 10.7409 minutes and exact mass of 296.12618. DON is a mycotoxin produced by fungus fusarium graminearum and causes the crop disease Fusarium Head Blight, a well recorded issue in Indiana at the time where the Lafayette meteorite was reportedly found. A DON standard was not run and at least 18 other isomers exist so we cannot be certain in this untargeted experiment that DON was detected. However the IDEOM algorithm used to identify metabolites from the chromatograms does predict the most likely isomer based on retention times, and it predicted DON as the most likely detected isomer. 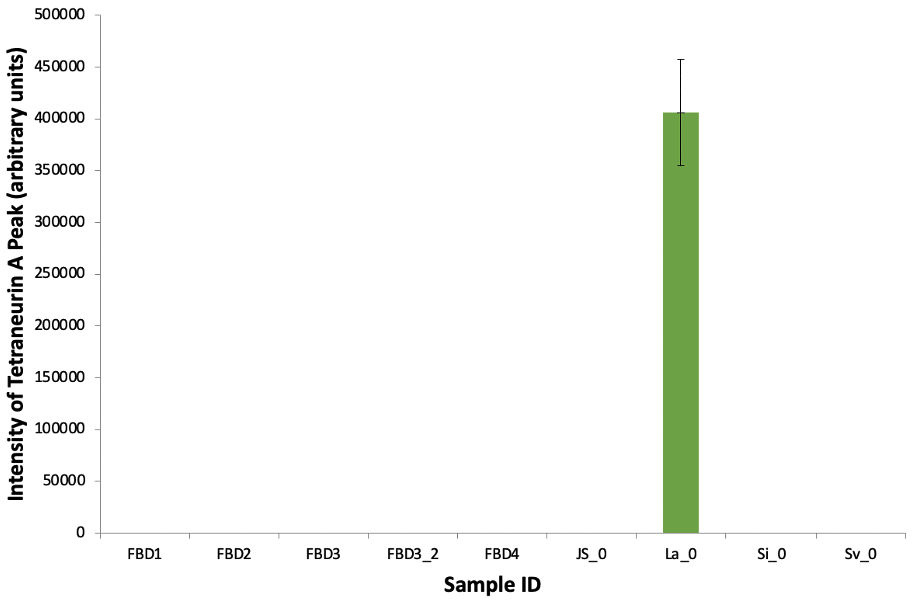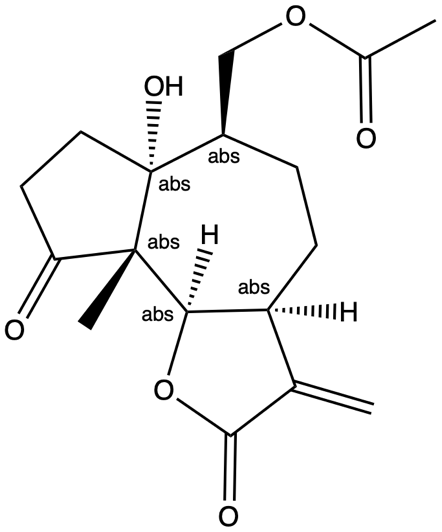 |
| 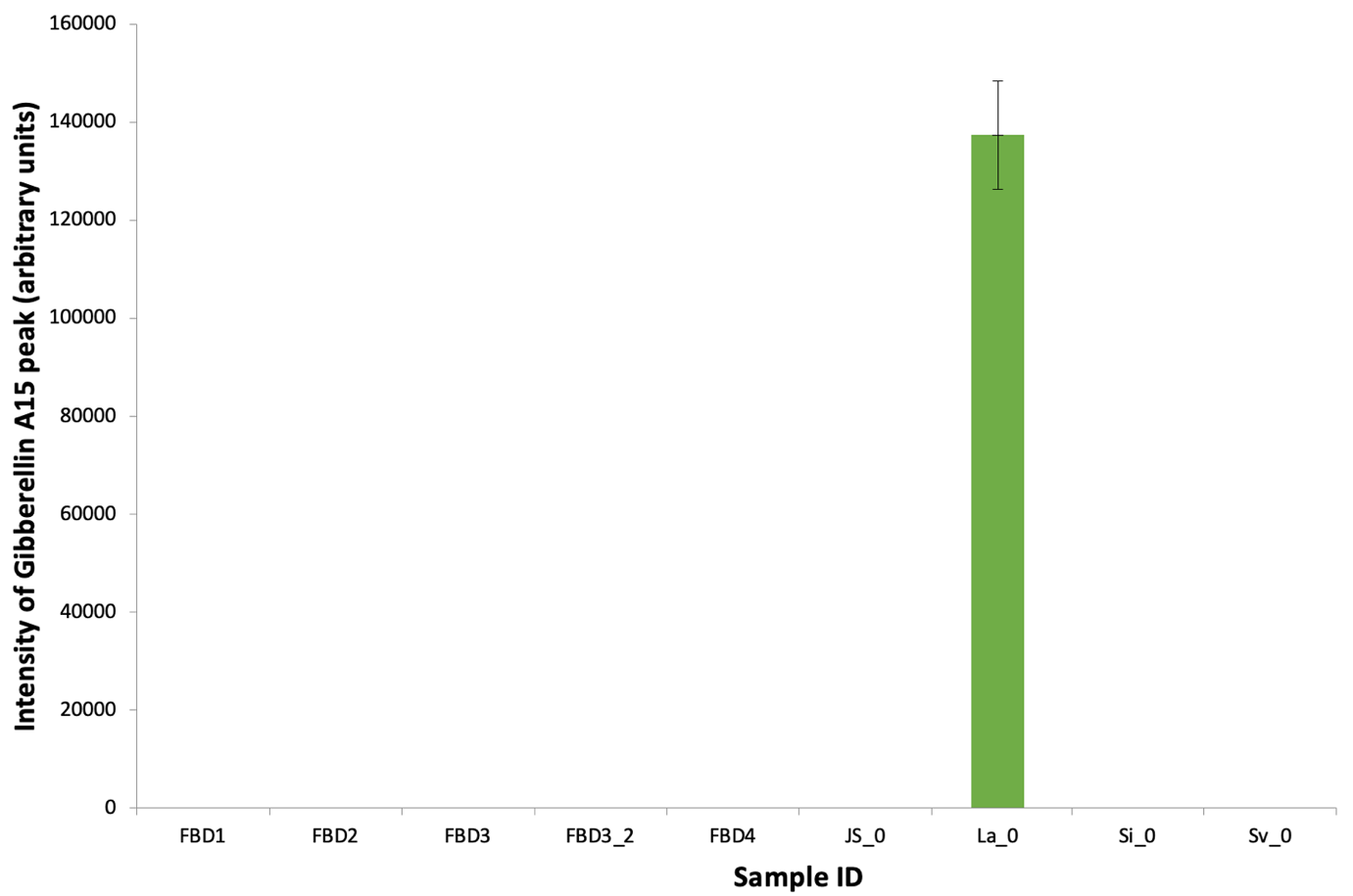 Figure SM 10 LCMS peak intensity distribution of metabolite putatively annotated as Gibberellin A15. This secondary metabolite was detected in negative mode with a retention time of 4.2260 minutes and had an exact mass of 330.1834. Gibberellin A15 is a terpenoid (akin to the sesquesterpene lactones above) produced by giberella fungi similar to DON produced by FHB, also a giberella produced secondary metabolite. 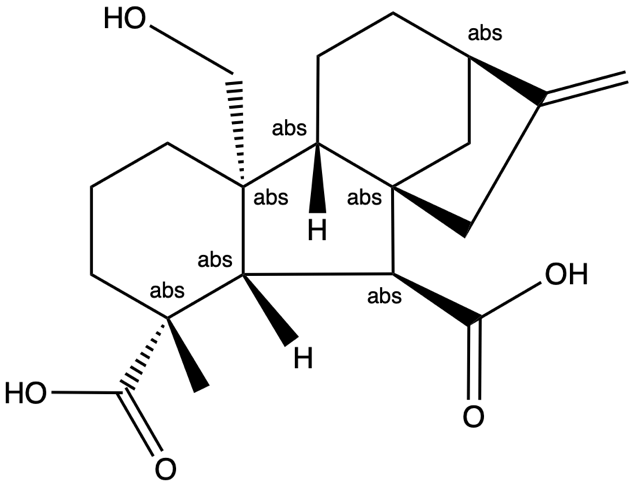 |
